# Biomarkers and acute brain injuries: interest and limits

**DOI:** 10.1186/cc13841

**Published:** 2014-04-24

**Authors:** Ségolène Mrozek, Julien Dumurgier, Giuseppe Citerio, Alexandre Mebazaa, Thomas Geeraerts

**Affiliations:** 1 Anesthesiology and Critical Care Department, Hopital Purpan, University Hospital of Toulouse, University Toulouse 3 Paul Sabatier, 1 place du Dr Baylac, 31059 Toulouse, France; 2 INSERM U942, University Paris Diderot, Sorbonne Paris Cite, CMRR Paris Nord-IDF, GH Saint Louis – Lariboisière – Fernand Widal, F-75010 Paris, France; 3 Neuroanaesthesia and Neurointensive Care Unit, Anestesia e Rianimazione, San Gerardo Hospital, via Pergolesi 33, 20900 Monza, Milan, Italy; 4 Department of Anesthesia and Intensive Care, INSERM U942, Paris Diderot University, Lariboisière Hospital, 2 rue Ambroise Paré, 75010 Paris, France

## Abstract

For patients presenting with acute brain injury (such as traumatic brain injury, subarachnoid haemorrhage and stroke), the diagnosis and identification of intracerebral lesions and evaluation of the severity, prognosis and treatment efficacy can be challenging. The complexity and heterogeneity of lesions after brain injury are most probably responsible for this difficulty. Patients with apparently comparable brain lesions on imaging may have different neurological outcomes or responses to therapy. In recent years, plasmatic and cerebrospinal fluid biomarkers have emerged as possible tools to distinguish between the different pathophysiological processes. This review aims to summarise the plasmatic and cerebrospinal fluid biomarkers evaluated in subarachnoid haemorrhage, traumatic brain injury and stroke, and to clarify their related interests and limits for diagnosis and prognosis. For subarachnoid haemorrhage, particular interest has been focused on the biomarkers used to predict vasospasm and cerebral ischaemia. The efficacy of biomarkers in predicting the severity and outcome of traumatic brain injury has been stressed. The very early diagnostic performance of biomarkers and their ability to discriminate ischaemic from haemorrhagic stroke were studied.

## Introduction

Despite significant advances in understanding the pathophysiology of brain injuries, there has been little change in terms of therapeutic or pharmacological treatment in recent years. The complexity and heterogeneity of lesions after brain injury are most probably responsible, at least in part, for the lack of positive results in clinical trials. Furthermore, patients with apparently comparable brain lesions on imaging may have different neurological outcomes or responses to therapy. The use of biomarkers in the setting of brain injury may be of interest not only for diagnosis and identification of intracranial lesions but also for the evaluation of the severity, prognosis and treatment efficacy. In addition, patient stratification, based on biomarkers, may be useful in clinical trials for selecting a homogeneous population and decreasing inclusion disparity.

Brain biomarker detection in the cerebrospinal fluid (CSF) and in the blood has been described. Due to the separation of the brain from the blood by the blood–brain barrier (BBB), proteins produced within the brain are present only in small quantities in the blood if the BBB is intact. The BBB status (open or closed) therefore has a strong influence on the amount of those types of proteins in the blood and must be taken into consideration for the interpretation of brain injury blood biomarkers.

The aim of this review is to summarise plasmatic and CSF biomarkers evaluated in subarachnoid haemorrhage (SAH), traumatic brain injury (TBI) and stroke, and to clarify their interest and limits for diagnosis and prognosis. Of note, the present review will not describe the neurological prognostic factors after cardiopulmonary resuscitation in patients with cardiac arrest. Serum levels of proteins neuron-specific enolase (NSE) and S100β are considered promising candidates for neurological predictors, and a review on the clinical usefulness of these markers has been published previously [1].

## Subarachnoid haemorrhage

### Initial severity and prognosis of subarachnoid haemorrhage

Several biomarkers have been studied in terms of the short-term or long-term neurological prognostic factors and correlation with initial severity of patients after aneurysmal SAH [2-13]. Table 1 summarises different biomarkers and their correlation with initial neurological patient severity and prognosis.

**Table 1:** Main biomarkers of subarachnoid haemorrhage, and dosage correlated with initial severity, neurological prognosis and mortality

	**Dosage**		**Initial severity**				**GOS**			
**Biomarker**	**CSF**	**Plasma**	**GCS**	**WFNS**	**HH**	**Fisher**	**3 months**	**6 months**	**12 months**	**Mortality**
ET-1	+	+			+ (CSF)					+ (plasma)
TNF-α	+	+			– CSF)		– (CSF)			
IL-6	+				–			+		
IL-1β	+				–		–			
ICAM-1, VCAM-1	+	+		– (CSF, plasma)	– (CSF, plasma)				– (CSF, plasma)	
Light-chain NF	+								+	
Heavy-chain NF	+		+	+			+			
ApoE	+		+			–	+			
S100β	+	+	– (CSF)	+ (plasma)		+ (plasma)	– (CSF)	+ (plasma)		
ANP		+			+ (plasma)	+ (plasma)				
BNP		+				+				+
cTnI		+	+		+	+	+			+
vWF, MMP-9, VEGF		+				+				
CRP	+	+	+ (CSF, plasma)		+ (CSF, plasma)	+ (CSF, plasma)	+ (CSF, plasma)			

+, correlation described; −, lack of correlation; plasma/CSF, dosing site. ANP, atrial natriuretic peptide; ApoE, apolipoprotein E; BNP, brain natriuretic peptide; cTnI, cardiac troponin I; CRP, C-reactive protein; CSF, cerebrospinal fluid; ET-1, endothelin-1; Fisher, Fisher classification; GCS, Glasgow Coma Scale; GOS, Glasgow Outcome Scale; HH, Hunt and Hunter classification; ICAM-1, intercellular adhesion molecule-1; IL, interleukin; MMP-9, matrix metalloproteinase-9; NF, neurofilament; S100β, S100β protein; TNF, tumour necrosis factor; VCAM-1, vascular cell adhesion molecule-1; VEGF, vascular endothelial growth factor; vWF, von Willebrand factor; WFNS, World Federation of Neurosurgeons classification.

### Vasospasm and cerebral ischaemia

Cerebral vasospasm and its related cerebral ischaemia remain the primary cause of mortality and neurological deficit after SAH and the most powerful predictors of long-term outcome [14,15]. Physiological and morphological changes observed during cerebral vasospasm occur in two phases: a contraction of the arterial wall in the first 72 hours after onset of SAH, followed by smooth muscle cell proliferation in the intima of the main cerebral arteries. Indeed, sustained arterial contraction causes an increase in the shear stress of endothelial cells, from day 3 to day 14 after SAH, with an increase in endothelial permeability, expression of intercellular adhesion molecules with intimal infiltration of leukocytes, platelet adhesion to the internal elastic lamina, migration of smooth muscle cells and myointimal proliferation [16,17]. Plasma and CSF biomarkers have been studied in the context of SAH, in relation to vasospasm and other factors such as systemic inflammation, microcirculatory disorders or microembolic release [18,19]. A recent review classified CSF biomarkers for cerebral vasospasm according to reports in the literature as markers with auspicious value, candidate markers with insufficient evidence and noncandidate markers with no reference to cerebral vasospasm [20].

#### Cytokines

An inflammatory response similar to that observed during coronary spasm appears to affect the cerebral circulation of patients with SAH. Proinflammatory cytokines – that is, IL-1β, IL-6 and tumour necrosis factor alpha – have been detected in the CSF of patients with SAH, with a peak between day 5 and day 9 followed by a gradual decrease [2]. Peak concentrations of cytokines have been found to be increased up to 10,000-fold, in the range detected in bacterial meningitis [21]. The concentrations of IL-1β and IL-6 are lower in the plasma than in the CSF, suggesting a cerebral origin of these mediators with a release mechanism.

The triggers for this marked inflammatory response in the subarachnoid space of patients with SAH are still unknown. One hypothesis is a complement activation method via osmotically induced disruption of erythrocytes [22,23]. A study of 35 patients with SAH revealed parallel changes in the velocities of the middle cerebral artery using transcranial Doppler and concentrations of IL-1β, IL-6 and tumour necrosis factor alpha in the CSF [2]. Another study of 64 SAH patients confirmed the increase in CSF IL-6 (peak at day 4 to day 5) before the onset of clinical signs of vasospasm (peak at day 6 to day 7), with a threshold of 2,000 pg/ml at day 4 for the prediction of the development of symptomatic vasospasm (sensitivity = 89% and specificity = 78%) [24]. Another recent study in 38 SAH patients reported higher concentrations of IL-6 in the CSF, brain extracellular fluid and plasma of symptomatic patients than in those of asymptomatic patients with vasospasm [25].

#### Natriuretic peptides

Atrial natriuretic peptide and brain natriuretic peptide (BNP) are produced in the heart in response to neural and humoral stimuli and fluid overload [26]. BNP is therefore not brain specific, but is also produced in brain tissue, especially in the hypothalamus. Two possible mechanisms for increased BNP production in the hypothalamus have been advanced: release secondary to humoral or paracrine signals, and a response to hypoxia due to vasospasm. Some evidence suggests that cerebral ischaemia after SAH is not only caused by large vessel spasm [27]. Many hypotheses to explain this phenomenon have been proposed, including systemic infarction, microcirculatory spasm, and the release of microemboli [18,19].

BNP may be a marker of a general process of microcirculatory dysfunction characterised by systemic inflammation and local thrombosis, as described in sepsis or haemorrhagic shock [28]. In addition, a recent study links BNP release to proinflammatory cytokines [29]. Berendes and colleagues [30] and Tomida and colleagues [31] reported an association between plasma BNP concentration and the development of delayed ischaemic neurological deficit (DIND). Sviri and colleagues [32] studied 38 patients with SAH and observed an increase in plasma BNP between day 1 and day 3 (69.6 ± 92.4 pg/ml) compared with control patients (5.8 ± 1.9 pg/ml). Patients not presenting with DIND have displayed a progressive decrease from day 3 in plasma BNP concentration. On the contrary, patients with DIND have displayed a gradual increase in plasma BNP concentration between day 3 and day 12 post-SAH [32]. A recent study of 119 patients revealed a significant association between a BNP level >276 pg/ml in the plasma and the onset of cerebral ischaemia [27]. Of note, BNP is biologically active and may increase the risk of cerebral ischaemia by its direct effects on the kidneys and systemic vessels, including natriuresis, vasodilatation and hypovolaemia.

#### von Willebrand factor, vascular endothelial growth factor and matrix metalloproteinase-9

During cerebral vasospasm, sustained arterial contraction is at the origin of increases in the shear stress of endothelial cells and is associated with modifications of endothelial permeability, expression of adhesion molecules and myointimal proliferation [16,17]. Vascular endothelial growth factor (VEGF) can initiate these changes because its concentration is increased in the intima after endothelial cell damage [33,34]. Matrix metalloproteinase-9 (MMP-9) alone can stimulate the activity of VEGF by increasing the availability of VEGF in the media of vessels [35]. Moreover, MMP-9 expression is increased in smooth muscle cells after alterations of endothelial cells, contributing to the initiation of myointimal proliferation [36].

von Willebrand factor is considered a plasma marker of endothelial cell injury. McGirt and colleagues have demonstrated an increase in plasma concentrations of VEGF, MMP-9 and von Willebrand factor before the diagnosis of vasospasm by both transcranial Doppler and cerebral angiography in 38 patients with SAH [13]. Peak concentrations were observed for von Willebrand factor, MMP-9 and VEGF at day 5, day 3 and day 2, respectively. Elevated plasma von Willebrand factor levels >5,500 ng/ml, MMP-9 levels >700 ng/ml and VEGF levels >0.12 ng/ml each independently increased the odds of vasospasm (17-fold, 25-fold and 21-fold, respectively). However, the plasma concentrations of these markers were not different between clinically symptomatic and asymptomatic patients with vasospasm. Recently, Chou and colleagues reported the lack of a correlation between CSF or plasmatic MMP-9 and vasospasm in 55 patients with SAH [5].

#### Endothelin-1

Endothelin-1 has major vasoconstrictive effects in human arteries, including cerebral vessels [37]. Furthermore, endothelin-1 has been found in neurons, glial cells, the choroid plexus and macrophages. The concentration of endothelin-1 in the CSF of SAH patients was significantly higher (2.5 ± 0.7 pg/ml) on the first day after onset of SAH than in the CSF of controls (normal values <0.85 pg/ml) [38]. Endothelin-1 concentrations in CSF increase until the sixth day and then gradually decrease in patients without vasospasm. In addition, a significant increase in CSF endothelin-1 has been observed between day 4 and day 7 in symptomatic patients with vasospasm [38,39]. One study found an endothelin-1 increase in the CSF before detection of angiographic vasospasm [38]. Moreover, a significant correlation has been found between the concentration of endothelin-1 in the CSF and the extension of angiographic vasospasm [40]. In plasma, no significant difference in endothelin-1 concentration has been demonstrated between patients with SAH and controls [41].

Endothelin receptor antagonists have emerged as a promising therapeutic option. A recent Cochrane database review concluded that endothelin receptor antagonists appear to reduce DIND and angiographic vasospasm, but their benefit to clinical outcome remains unproven. Moreover, their associated adverse events were not negligible (for example, hypotension and pneumonia) [42].

#### Intercellular adhesion molecule-1 and vascular cell adhesion molecule-1

There is a large amount of evidence that inflammatory reactions may be involved in the pathogenesis of delayed ischaemic lesions. Several molecules could initiate the steps of the inflammatory cascade. These include intercellular adhesion molecule-1, an immunoglobulin-like molecule that is exposed to endothelial cells and induced by exposure to inflammatory cytokines, and vascular cell adhesion molecule-1 [43,44]. Animal studies have demonstrated an upregulation of intercellular adhesion molecule-1 on endothelial and medial layers of cerebral arteries after SAH. Treatment with monoclonal antibodies against intercellular adhesion molecule-1 can reduce or even inhibit cerebral vasospasm in animals [45]. An increase in the blood and CSF concentrations of intercellular adhesion molecule-1 and vascular cell adhesion molecule-1 in patients with SAH compared with a control group within the first 7 days has been described [7]. There appears to be a correlation between cerebral blood flow velocities measured using transcranial Doppler and a secondary increase of intercellular adhesion molecule-1 and vascular cell adhesion molecule-1 in plasma and CSF [46].

#### Neurofilaments

Neurofilaments are components of the axonal cytoskeleton and include heavy-chain neurofilaments (NF-H; 190 to 210 kDa), medium-chain neurofilaments (160 kDa), light-chain neurofilaments (68 kDa) and α-internexin (66 kDa) [47]. In physiological conditions, neurofilaments are restricted to the intracellular compartment of the neuronal cells. Alteration of the axonal membrane integrity can result in the release of neurofilament proteins in the extracellular space and their spread into the CSF. The subunits of neurofilaments are therefore potentially useful for revealing axonal injury.

Plasma NF-H concentrations in healthy individuals average 0.11 ng/ml, and CSF NF-H concentrations average 0.94 ng/ml [48]. Petzold and colleagues reported a positive correlation between CSF concentrations of NF-H and prognosis (Glasgow Outcome Scale at 3 months) in SAH patients [49]. Lewis and colleagues confirmed that high concentrations of NF-H in plasma and CSF were associated with a poor outcome at 6 months and that patients with vasospasm had increased levels of NF-H in CSF and plasma (16.7 ± 19.9 ng/ml and 0.44 ± 0.68 ng/ml) compared with patients without vasospasm (0.29 ± 0.44 ng/ml and 8.3 ± 15.3 ng/ml) [9]. NF-H may thus be a useful marker of axonal injury in SAH.

More recently, Zanier and colleagues [41] reported higher concentrations of light-chain neurofilaments in CSF obtained by external ventricular shunt in patients with early cerebral ischaemia defined by hypodense lesion on computed tomography (CT) within 72 hours of ruptured aneurysm (related to intracranial haemorrhage or complications of aneurysm treatment). However, there were no significant differences in external ventricular shunt light-chain neurofilaments concentrations between patients who developed clinical vasospasm and those with delayed cerebral ischaemia [41].

#### α2-spectrin breakdown products

α2-spectrin is a cytoskeletal protein. The products of its degradation by calpain and caspase-3 are potential markers of the severity of lesions in SAH. α2-spectrin is transformed into degradation products of 150 kDa (SBDP150) and 145 kDa (SBDP145) by calpain and is cleaved into a degradation product of 120 kDa (SBDP120) by caspase-3 [50]. Calpain and caspase-3 are major effectors of cell death (respectively, necrotic and apoptotic). In a study of 20 patients with a high Fisher grade of SAH, Lewis and colleagues reported an increase in SBDP concentration in the CSF [51]. SBDP150, SBDP145 and SBDP120 CSF concentrations were higher in patients with clinical vasospasm compared with patients who did not develop vasospasm. Moreover, symptomatic vasospasm was associated with an increase in the concentrations of SBDPs (SBDP145 and SBDP150) in the CSF 12 hours prior. The treatment of vasospasm induced a decrease in SBDPs to baseline levels in patients without ischaemia, but SBDP concentrations remained high in patients with cerebral ischaemia.

#### S100β protein

S100β protein belongs to a multigenic family of low molecular weight (9 to 13 kDa) calcium-binding S100 proteins. S100β protein is mainly expressed in glial cells, particularly astrocytes [52]. S100β protein is involved in intracellular signal transduction via the inhibition of protein phosphorylation, regulation of enzyme activities and affecting calcium homeostasis [53]. In addition, S100β protein participates in the regulation of cell morphology by interacting with elements of the cytoplasmatic cytoskeleton. S100β protein is actively secreted into the CSF from astrocytes and is believed to have extracellular functions. The protein can be detected in both CSF (normal value 1 to 2 μg/l) and blood serum (normal value <0.15 μg/l), resulting from the elimination process after intracellular and extracellular actions. S100β protein’s biological half-life is 2 hours; the protein can be detected in both CSF and blood serum. Kay and colleagues report an increase (compared with a control population) of its concentration in CSF after SAH in patients with neurologic symptoms [10]. A recent study of 55 patients with SAH shows that plasma and CSF concentrations of S100β can detect cerebral ischaemia and intracranial hypertension after SAH, a secondary increase in plasma concentration being predictive of vasospasm [54].

#### Other biomarkers

Recently, Siman and colleagues [55] studied combinations of neurodegeneration biomarkers for predicting vasospasm, infarction and outcome rather than the use of a single biomarker. They reported an increase for six CSF biomarkers from 3-fold to 10-fold between days 1 and 5 after SAH onset for patients with moderate to severe angiographic vasospasm (14-3-3β protein, 14-3-3ζ protein, ubiquitin C-terminal hydrolase-L-1 (UCH-L1), NSE and two SBDPs cleaved by calpain). These biomarkers were correlated significantly with occurrence of cerebral vasospasm, brain infarction and poor outcome. They reported the 14-3-3β protein, NSE and fragment N-terminal of SBDPs as early predictors of vasospasm [55].

In clinical practice, none of these biomarkers have been clearly validated for the early detection of cerebral vasospasm, the main cause of mortality and neurological deficit after SAH. Larger and prospective studies are required to validate their use for detection of vasospasm, but also to validate therapeutic options guided by biomarker levels aiming at improving neurological outcome.

## Traumatic brain injury

TBI severity can be assessed using the Glasgow Coma Score (GCS) and brain imaging. Minor TBI (GCS 13 to 15) and moderate TBI (GCS 9 to 12) represent 90% of TBI cases, but these types of TBI may induce long-term sequelae. Because of the limits of GCS and imaging, the use of biomarkers to improve diagnosis and classification of TBI could be of interest.

### Initial severity, prognosis and mortality

Many biomarkers have been studied in TBI to evaluate the association of initial severity with the GCS and neuroradiological findings at patient admission, neurologic outcome predictions with Glasgow Outcome Scale (GOS) at 3 months and 6 months, and mortality prediction. Several biomarkers have been found to correlate with these associated items: S100β protein [56,57], NSE [58,59], UCH-L1 [60-62], glial fibrillary acidic protein (GFAP) [57,58], myelin basic protein [63,64] and tau protein [65] in plasma, and S100β protein [56], UCH-L1, SBDPs [66,67] and tau protein [68] in CSF.

### Classification of traumatic brain injury

A recent review summarised CSF and blood biomarkers of mild TBI to predict long-term neurological sequelae and to assess patients with head trauma by classifying them according to axonal, neuronal or astroglial injuries [69].

#### S100β protein

S100β protein can be released from astroglial cells in many ways: by activation of adenosine and glutamate receptors [70], by stimulation of astroglial 5HT1A receptors [71] and by adrenocorticotropic hormone and corticotrophin-like intermediate-lobe peptide [72]. Moreover, S100β protein is secreted from proliferating astrocytes.

In TBI patients, the acute increase in plasma S100β protein level is most probably related to massive adenosine and glutamate release in heavily damaged and perfused brain areas [73]. A portion of S100β protein is able to diffuse into the bloodstream. The determination of plasma S100β protein after TBI may be able to differentiate groups of patients with minor or severe injuries. In 226 patients with minor TBI (GCS 13 to 15), the plasma levels of S100β were significantly higher in patients with intracranial injury, with a threshold value of 0.10 μg/l for detecting lesions on CT scan (area under receiver operating characteristic curve = 0.73 (95% CI = 0.62 to 0.84) and sensitivity = 95%) [74]. For 2,128 patients with minor TBI, the plasma threshold was 0.12 μg/l, with a sensitivity of 99% and a specificity around 20% for the detection of intracranial lesions on CT scan. The negative predictive value was 99.7% (95% CI = 98.1 to 100%) [75]. A S100β protein level below 0.12 μg/l at patient admission could therefore be used to exclude post-traumatic intracranial lesions on CT scan. However, these data require confirmation in a larger study. S100β protein, initially considered to be located only in the central nervous system, is expressed in other tissues such as adipocytes or chondrocytes. High plasma protein S100β has been observed after multiple traumas in patients without brain damage, leading to questioning of its usefulness for predicting neurological outcome in those patients [76].

Goyal and colleagues [56] studied S100β protein temporal profiles in the CSF and plasma of adults with severe TBI. Their temporal serum profiles were associated with acute mortality, perhaps because of extracerebral sources in the serum as represented by high Injury Severity Scores, but the CSF S100β protein profiles were associated with outcomes and mortality [56]. In clinical practice, the S100β protein level can be obtained in 1 hour and its cost is approximately €15.

#### Neuron-specific enolase

NSE is one of the five isoenzymes of glycolytic enolase in central and peripheral neurons. NSE is localised in neuron cytoplasm and is most probably involved in the increase of chloride concentration at the beginning of neural activity [77]. This marker has been used to evaluate neuronal functional alterations. NSE is passively released rapidly in the plasma after TBI by cell destruction. The NSE plasma concentration at patient admission for TBI has been found to be twofold higher than normal reference values [58]. Despite these promising data, several studies have produced disappointing results. Because of the slow elimination (biological half-life of 48 hours) of NSE from the plasma, quantification of the amount of brain injury and distinction between primary and secondary insult remains difficult using plasma NSE [78]. Furthermore, NSE can be released into the plasma from red blood cell haemolysis, resulting in possible confounding factors [79].

#### Ubiquitin C-terminal hydrolase-L-1

UCH-L1 is highly and specifically expressed in neurons. UCH-L1 represents approximately 1 to 5% of the total soluble proteins within the brain [80]. This protein is involved in the addition and deletion of ubiquitin-dependent protein (via the ATP-dependent proteasome pathway), playing an important role in the removal of excessive, oxidised or abnormal proteins during normal and neuropathological conditions [81]. One study reports higher concentrations of UCH-L1 in the CSF of patients with severe TBI (44.2 ng/ml) compared with a control group (2.7 ng/ml) [60]. UCH-L1 is released within 6 hours after trauma and peaks in the first 24 hours in the CSF. This study reported an area under the curve (AUC) of 0.88 (95% CI = 0.68 to 1.00) using UCH-L1 CSF levels within the first 6 hours versus control patients.

UCH-L1 appears to be able to distinguish TBI and uninjured control patients at 6 hours when the mental status can be confounded by drugs, alcohol or other pathology. Recently, Papa and colleagues [82] compared early UCH-L1 plasma levels (within 4 hours of injury) of patients with mild and moderate TBI with uninjured and injured control patients in a prospective cohort study. They reported a significant difference between UCH-L1 levels in CT-negative patients versus CT-positive patients (0.62 ng/ml vs. 1.61 ng/ml, respectively) with an AUC of 0.73 (95% CI = 0.62 to 0.84). Moreover, UCH-L1 levels allow one to distinguish mild and moderate TBI from uninjured control patients with an AUC of 0.87 (95% CI = 0.82 to 0.92) and to distinguish TBI with GCS 15 from controls with an AUC of 0.87 (95% CI = 0.81 to 0.93) [82].

#### Glial fibrillary acidic protein

GFAP is a protein involved in astrocyte cytoskeletons by forming networks with filaments that provide support and strength to cells. Glial cells specifically express GFAP, which is involved in several neurological processes such as BBB integrity. An increase in the plasma concentrations of GFAP in patients with severe TBI (0.10 ± 0.18 μg/l on admission, 0.012 ± 0.026 μg/l 24 hours after injury and 0.017 ± 0.052 μg/l 48 hours after injury) has been reported compared with healthy volunteers (0.004 μg/l) [83].

Moreover, critically injured trauma patients without TBI had significantly lower levels of plasmatic GFAP compared with patients with TBI documented on head CT scan [84]. In addition, the plasma concentration of GFAP is not affected by multiple traumas without brain injury [85]. GFAP has recently been reported as highly vulnerable to proteolytic modifications *in vitro* and *in vivo*. Breakdown products of GFAP are therefore likely to be present in biofluids. GFAP breakdown product levels are able to differentiate TBI patients from uninjured controls with an AUC of 0.90 (95% CI = 0.86 to 0.94) and differentiate TBI patients with a GCS of 15 from normal controls with an AUC of 0.88 (95% CI = 0.82 to 0.93) [86]. More recently, the prospective Transforming Research and Clinical Knowledge in TBI study evaluated the diagnosis accuracy of elevated levels of GFAP breakdown products in TBI patients. This study confirms the good correlation between GFAP breakdown product levels and CT scan findings in TBI patients [87].

#### α2-spectrin breakdown products

Pineda and colleagues reported an increase in SBDP concentration in the CSF after severe TBI [66]. More recently, Mondello and colleagues [67] studied 40 severe TBI patients using SBDP measurement in the CSF from ventriculostomy catheters every 6 hours for a maximum of 7 days following TBI, comparing them with control patients. Compared with control patients, both SBDP145 (14.42 ± 0.91 ng/ml vs*.* 0.52 ± 0.22 ng/ml) and SBDP120 (6.05 ± 0.28 ng/ml vs*.* 1.21 ± 0.48 ng/ml) CSF concentrations were increased in severe TBI. The degradation of products appears to be different, with an earlier peak for SBDP145 (29.56 ng/ml at 6 hours) compared with a late peak for SBDP120 (11.96 ng/ml at 138 hours). These observations suggest that cell death via necrosis or apoptosis is activated with a different time course after severe TBI. In addition, patients who died after TBI exhibited higher concentrations of SBDP145 and SBDP120 than survivors within 7 days post-trauma [67].

In clinical practice, only S100β protein may be used to screen patients with minor TBI (GCS 13 to 15) and exclude CT-scan lesions when the plasma level is below 0.12 μg/l at admission. UCH-L1 may have the same utility but prospective studies with larger samples are required. GFAP has the advantage of not being influenced by peripheral injuries, contrary to S100β protein and NSE, and is therefore probably more specific for brain injury [88]. The use of biomarkers for classification of TBI is certainly of major interest, but large clinical studies validating strategies based on biomarkers use in TBI are still lacking, particularly in severe TBI patients.

## Stroke

The use of biomarkers to diagnose stroke very early and the precise extent of brain damage may be useful in the application of specific therapeutic strategies. The difficulty with this approach relates to the heterogeneity of the brain cell population, different tolerances to ischaemia and distribution in the central nervous system, complexity of the ischaemic cascade and integrity of the BBB. Biomarkers may also reflect the different steps of cerebral ischaemia, such as inflammation, glial activation and neuronal injury.

### S100β protein

Several studies have described a significant increase in plasma levels of S100β protein within the first 3 days after cerebral infarction [89,90]. In stroke, high levels of adenosine occur in the core of the infarct, not perfused with blood. S100β protein accumulated in this region cannot be released into the bloodstream and thus does not contribute to any observed increase in plasma levels. The pattern of reactive astrogliosis observed in animals and human studies explains the plasma S100β protein temporal profiles in stroke patients, with plasma S100β protein peaking later than in TBI patients. A recent review described the serum S100β temporal profile after stroke onset. There is a gradual concentration increase starting 8 to 10 hours after onset of symptoms, followed by a peak at 72 hours and then a drop at 96 hours [91]. Lower plasma concentrations of S100β have been reported in only one study, in patients with transient ischaemic attack (TIA) or normal brain CT on admission in comparison with individuals with neurological deficits or abnormal brain imaging displaying cortical infarcts [73]. A correlation has been observed between plasma levels of S100β protein and the size of cerebral infarction [92,93]. An association has been described between S100β protein plasma levels and the National Institutes of Health Stroke Score [89,94]. However, the delayed kinetics and low specificity preclude this association for diagnostic use in acute stroke situations. The increase in plasma S100β is not specific for cerebral infarction and can be observed with other neurological conditions such as TBI and extracranial malignancies, possibly leading to biased interpretations of results. The clinical performance of S100β protein therefore does not appear to be robust enough to differentiate ischaemic stroke, haemorrhagic stroke and stroke mimics.

Despite these factors, S100β concentrations could be an additional tool for the identification of patients at high risk of specific early neurological complications in clinical practice. Indeed, a plasmatic S100β level >1.03 μg/l at 24 hours after the onset of stroke predicts malignant infarction in patients with proximal middle cerebral artery occlusion, with a sensitivity of 94% and a specificity of 83% [95]. Another study has reported higher S100β prethrombolysis concentrations in patients who developed haemorrhagic transformation after thrombolysis treatment compared with patients who did not (0.14 vs*.* 0.11 μg/l) [96]. A recent study examined 458 patients with ischaemic stroke who were not treated with thrombolytic drugs. At admission, patients with clinical deterioration caused by haemorrhagic transformation had higher concentrations of S100β and tight-junction proteins, which are markers of BBB breakdown. An analysis of these proteins levels could be used to screen for and predict the risk of haemorrhagic transformation [97].

### Asymmetric dimethylarginine

Methylarginines are synthesised by post-translational methylation of l-arginine and are released as free dimethylarginines after proteolysis. Asymmetric dimethylarginine (ADMA) and symmetric dimethylarginine are detectable in blood, urine and CSF. Whereas symmetric dimethylarginine is inactive, ADMA is a potential inhibitor of nitric oxide synthase, which is involved in endothelial dysfunction [98]. An increase in the ADMA plasma concentration is thus assumed to be a surrogate marker for the risk of ischaemic stroke.

Yoo and Lee reported a significant difference between ADMA plasma concentrations in healthy control patients (0.93 ± 0.32 μmol/l), ischaemic stroke patients (1.46 ± 0.77 μmol/l) and patients with initial recurrence of ischaemic stroke (2.28 ± 1.63 μmol/l) [99]. Another study that included 880 women revealed that an increase of 0.15 μmol/l ADMA in plasma leads to a 30% increased risk of ischaemic stroke and myocardial infarction [100]. In addition, the Framingham Offspring Study evaluated plasma ADMA concentrations from 2,013 individuals for whom simultaneous neuroimaging studies were available. The ADMA concentration was independently associated with an increased prevalence of magnetic resonance imaging abnormalities in the absence of clinical symptoms, which is a well-known risk factor for pre-emptive stroke [101].

### Matrix metalloproteinase-9

Matrix metalloproteinases are a family of zinc-dependent and calcium-dependent endopeptidases responsible for turnover and degradation of extracellular matrix proteins. The expression of MMP-9 in brain tissue under normal conditions is very low, but increases in MMP-9 expression have been demonstrated in ischaemic brain tissue [102]. The upregulation of MMP-9 occurs in brain tissue in response to injury and is believed to play a central role in the pathophysiology of ischaemic stroke by degradation of extracellular matrix proteins. After the onset of cerebral ischaemia, the uncontrolled expression and activity of MMP-9 mediate proteolysis and lead to BBB leakage and cell death.

Increases of MMP-9 plasma concentrations have been demonstrated in both ischaemic (149.6 ± 99 ng/ml) and haemorrhagic stroke patients upon presentation to the emergency department compared with healthy individuals (<97 ng/ml), suggesting a relatively short time period (within hours) from release to detection in the plasma [103,104]. Plasma concentrations of MMP-9 are also related to cerebral infarction size, neurological outcomes and haemorrhagic transformation, especially after fibrinolysis [104-106]. At patient admission to the hospital, plasma concentrations of MMP-9 are predictive of cerebral infarct volume on magnetic resonance imaging and are correlated with stroke lesion growth, even after thrombolysis administration [107].

### *N*-methyl-d-aspartic acid receptor antibodies and peptides

Receptors for *N*-methyl-d-aspartic acid bind the glutamate neurotransmitter and are expressed mainly by neuronal cells. The receptors contain four subunits (two NR1 and two NR2 subunits), and fragmentation of NR2 into NR2A and NR2B peptides is thought to occur during cerebral ischaemia or neurotoxicity [108,109]. The generation of *N*-methyl-d-aspartic acid receptor antibodies (NR2Abs) is mediated by the immune response following ischaemic events. The NR2Abs and NR2 peptides can be assayed in blood and CSF.

Several studies have examined the potential usefulness of NR2Abs and NR2 peptides as markers of ischaemic stroke. Dambinova and colleagues reported an increase in NR2Ab plasma concentrations during ischaemic stroke (5.01 ± 1.23 μg/l) and TIA (4.02 ± 2.04 μg/l) in 105 patients compared with 255 control subjects (1.49 ± 0.22 μg/l) [110]. NR2Abs are not able to discriminate stroke from TIA. Moreover, the NR2Ab increase is not observed after haemorrhagic stroke, suggesting that a negative NR2Ab result could be used to rule out haemorrhagic stroke. A threshold ≥2 μg/l has a sensitivity of 97% and a specificity of 98% in the diagnosis of ischaemic stroke or TIA within 3 hours after symptom onset. An increase of antibodies can be observed in hypertensive patients and in patients with a history of ischaemic stroke or atherosclerosis [110]. Thus, it is unclear whether the increase in antibody level reflects an acute episode of cerebral ischaemia or is a potential predictor of cerebrovascular events. A prospective multicentre study of 557 patients undergoing coronary surgery reported that 24 of 25 patients with a preoperative concentration NR2Ab ≥2 μg/l revealed neurologic complication within 48 hours after surgery [111].

### Glial fibrillary acidic protein

Clinical studies have demonstrated an increase in GFAP plasma levels after ischaemic stroke compared with control subjects, with a peak between day 2 and day 4 after onset of symptoms [112]. A prospective study involving 135 patients admitted 6 hours after onset of stroke symptoms reported detection of serum GFAP in 81% of patients with haemorrhagic stroke but in only 5% of those with ischaemic stroke [113]. Furthermore, plasma levels of GFAP were significantly higher in haemorrhagic stroke patients (mean value 111.6 ng/l) than in ischaemic stroke patients (mean value 0.4 ng/l). With a threshold value of 2.9 ng/l, the sensitivity was 79% and the specificity was 98% for differentiating ischaemic stroke from haemorrhagic stroke. In a study by the same team, the optimal timing to differentiate cerebral ischaemia from haemorrhage with GFAP was 2 to 6 hours after symptom onset [114]. A multicentre study focusing on S100β protein, NSE, GFAP and activated protein C–protein C inhibitor complex demonstrated the ability of GFAP to differentiate haemorrhagic stroke from ischaemic stroke, which has not been observed for other proteins [115]. Moreover, the combination of GFAP with activated protein C–protein C inhibitor complex and the National Institutes of Health Stroke Score led to a diagnostic sensitivity and negative predictive value of 100%, allowing exclusion of haemorrhagic stroke, which is potentially useful for early initiating fibrinolysis.

### Neuropeptide proenkephalin A and protachykinin

Stroke has been characterised by biomarkers of infarct size and damage to the BBB. Recent studies have reported stable precursor fragments of the neuropeptides encephalin (proenkephalin A (PENK-A)) and substance P (protachykinin A) as potent markers of BBB integrity [116]. Both neuropeptides are active as neurotransmitters and are involved in nociception and immune stimulation. Doehner and colleagues [117] recently evaluated PENK-A and protachykinin A in 189 patients presenting with symptoms of acute cerebrovascular disease. Plasma concentrations of PENK-A were significantly increased in acute stroke patients (123.8 pmol/l) compared with patients with TIA (114.5 pmol/l) or nonischaemic events (102.8 pmol/l). The elevation of PENK-A was correlated with stroke severity (National Institutes of Health Stroke Score) and with CT infarct size. Moreover, increased PENK-A concentrations predicted 3-month outcomes for mortality, stroke recurrence and myocardial infarction. Protachykinin A concentrations did not demonstrate any discriminative power [117].

### Other biomarkers and biomarker combinations

Several other biomarkers, mostly nonspecific, were studied either alone or in combination in the context of stroke. Combinations of several biomarkers have been developed to increase the sensitivity and specificity of the diagnosis [118-121].

In clinical practice, the main interest for stroke biomarkers is probably in the ability to discriminate ischaemic strokes from haemorrhagic strokes or TIA, allowing an early initiation of fibrinolysis. When taking into consideration the specificity for ischaemic event detection and the kinetics for biomarker increase, plasma PENK-A seems to be one of the most interesting biomarkers for acute ischaemic stroke detection.

**Figure 1:**
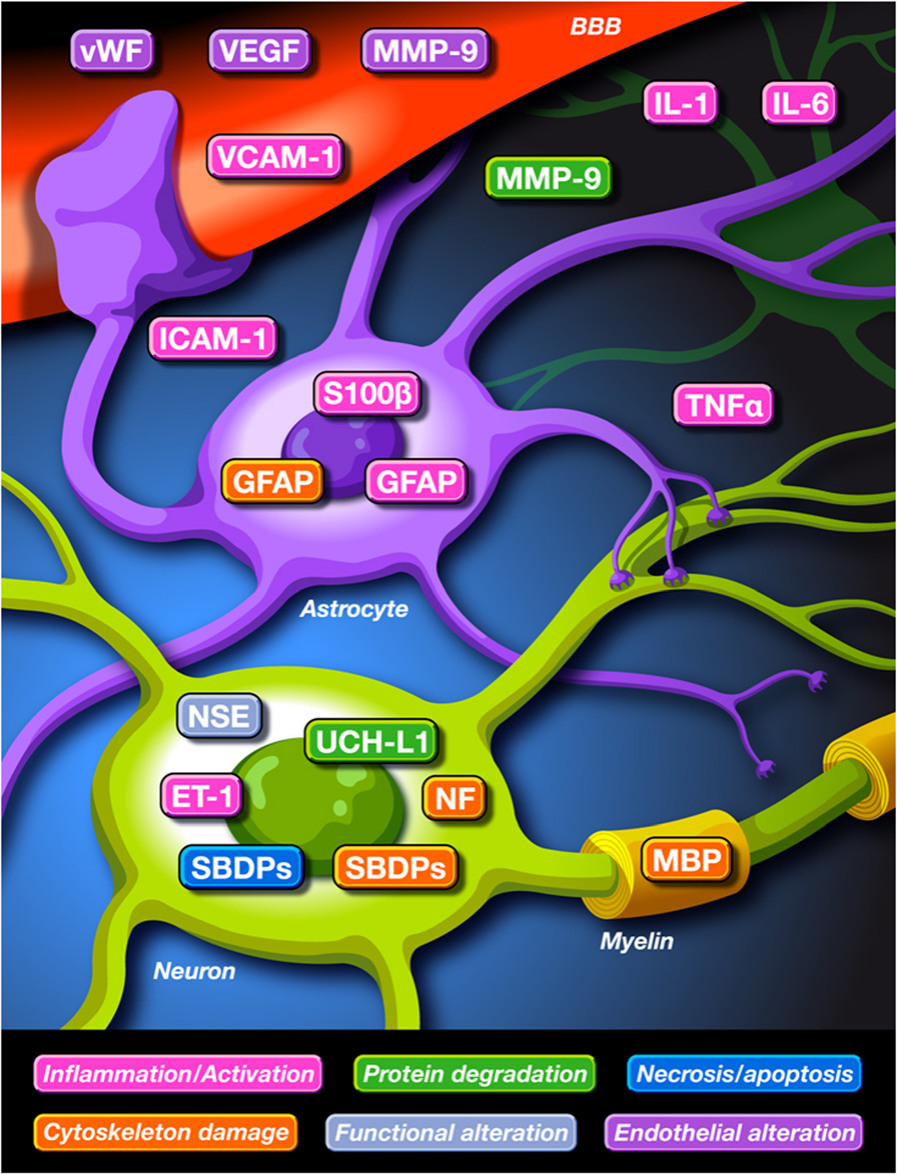
**Main biomarkers used in subarachnoid haemorrhage, traumatic brain injury and stroke.** Biomarkers can be classified according to their role in brain injuries: inflammation and activation; protein degradation; necrosis and apoptosis; cytoskeleton damage; functional alteration; and endothelial alteration. BBB, blood–brain barrier; ET-1, endothelin-1; GFAP, glial fibrillary acidic protein; ICAM-1, intercellular adhesion molecule-1; IL, interleukin; MBP, myelin basic protein; MMP-9, matrix metalloproteinase-9; NF, neurofilament; NSE, neuron-specific enolase; S100β, S100β protein; SBDP, α2-spectrin breakdown product; TNF, tumour necrosis factor; UCH-L1, ubiquitin C-terminal hydrolase-L-1; VCAM-1, vascular cell adhesion molecule-1; VEGF, vascular endothelial growth factor; vWF, von Willebrand factor.

Figure 1 summarises the main biomarkers examined in SAH, TBI and stroke. They are classified according to their significance in brain injury dynamics.

## Conclusion

The use of biomarkers in the treatment of brain injuries and brain diseases is of considerable interest for improving diagnosis and prognostication. These surrogate markers must nevertheless be used with caution. *Stricto sensu*, their performance at predicting an event can be applied only to populations in which they have been validated. The overuse of biomarkers for brain injuries could induce both expensive and counterproductive strategies. However, it appears reasonable to limit their use for clinical research.

Research on biomarkers of brain injury should remain a strong priority, as biomarkers could be a key factor in personalised medicine. New developments such as omics tools should be used in stroke treatment and therapy, similar to how they have been used recently in cardiovascular disease [122]. In parallel with the discovery of new biomarkers of brain injury, the economic performance of these biomarkers needs to be evaluated in both large cohorts of patients and in selected and targeted populations with complicated clinical situations and high uncertainty.

## Abbreviations

ADMA: Asymmetric dimethylarginine; AUC: Area under the curve; BBB: Blood–brain barrier; BNP: Brain natriuretic peptide; CI: Confidence interval; CSF: Cerebrospinal fluid; CT: Computed tomography; DIND: Delayed ischaemic neurological deficit; GCS: Glasgow coma score; GFAP: Glial fibrillary acidic protein; IL: Interleukin; MMP-9: Matrix metalloproteinase-9; NF-H: Heavy-chain neurofilaments; NR2Ab: *N*-methyl-d-aspartic acid receptor antibody; NSE: Neuron-specific enolase; PENK-A: Proenkephalin A; SAH: Subarachnoid haemorrhage; SBDP: α2-spectrin breakdown product; TBI: Traumatic brain injury; TIA: Transient ischaemic attack; UCH-L1: Ubiquitin C-terminal hydrolase-L-1; VEGF: Vascular endothelial growth factor.

## Competing interests

The authors declare that they have no competing interests.

## References

[B1] Shinozaki K Oda S Sadahiro T Nakamura M Hirayama Y Abe R Tateishi Y Hattori N Shimada T Hirasawa H S-100B and neuron-specific enolase as predictors of neurological outcome in patients after cardiac arrest and return of spontaneous circulation: a systematic review Crit Care 2009 13 R121 10.1186/cc7973 19624826 PMC2750170

[B2] Fassbender K Hodapp B Rossol S Bertsch T Schmeck J Schutt S Fritzinger M Horn P Vajkoczy P Kreisel S Brunner J Schmiedek P Hennerici M Inflammatory cytokines in subarachnoid haemorrhage: association with abnormal blood flow velocities in basal cerebral arteries J Neurol Neurosurg Psychiatry 2001 70 534 537 10.1136/jnnp.70.4.534 11254783 PMC1737308

[B3] Weiss N Sanchez-Pena P Roche S Beaudeux JL Colonne C Coriat P Puybasset L Prognosis value of plasma S100B protein levels after subarachnoid aneurysmal hemorrhage Anesthesiology 2006 104 658 666 10.1097/00000542-200604000-00008 16571959

[B4] Fountas KN Tasiou A Kapsalaki EZ Paterakis KN Grigorian AA Lee GP Robinson JS Jr Serum and cerebrospinal fluid C-reactive protein levels as predictors of vasospasm in aneurysmal subarachnoid hemorrhage, Clinical article Neurosurg Focus 2009 26 E22 10.3171/2009.2.FOCUS08311 19409001

[B5] Chou SH Feske SK Simmons SL Konigsberg RG Orzell SC Marckmann A Bourget G Bauer DJ De Jager PL Du R Arai K Lo EH Ning MM Elevated peripheral neutrophils and matrix metalloproteinase 9 as biomarkers of functional outcome following subarachnoid hemorrhage Transl Stroke Res 2011 2 600 607 10.1007/s12975-011-0117-x 22207885 PMC3236293

[B6] Witkowska AM Borawska MH Socha K Kochanowicz J Mariak Z Konopka M TNF-alpha and sICAM-1 in intracranial aneurismal rupture Arch Immunol Ther Exp (Warsz) 2009 57 137 140 10.1007/s00005-009-0010-4 19340565 PMC2771136

[B7] Kaynar MY Tanriverdi T Kafadar AM Kacira T Uzun H Aydin S Gumustas K Dirican A Kuday C Detection of soluble intercellular adhesion molecule-1 and vascular cell adhesion molecule-1 in both cerebrospinal fluid and serum of patients after aneurysmal subarachnoid hemorrhage J Neurosurg 2004 101 1030 1036 10.3171/jns.2004.101.6.1030 15597765

[B8] Kessler IM Pacheco YG Lozzi SP de Araujo AS Jr Onishi FJ de Mello PA Endothelin-1 levels in plasma and cerebrospinal fluid of patients with cerebral vasospasm after aneurysmal subarachnoid hemorrhage Surg Neurol 2005 64 Suppl 1 S1:2 S1:5 discussion S1:5 15967223 10.1016/j.surneu.2005.04.014

[B9] Lewis SB Wolper RA Miralia L Yang C Shaw G Detection of phosphorylated NF-H in the cerebrospinal fluid and blood of aneurysmal subarachnoid hemorrhage patients J Cereb Blood Flow Metab 2008 28 1261 1271 10.1038/jcbfm.2008.12 18319731

[B10] Kay A Petzold A Kerr M Keir G Thompson E Nicoll J Decreased cerebrospinal fluid apolipoprotein E after subarachnoid hemorrhage: correlation with injury severity and clinical outcome Stroke 2003 34 637 642 10.1161/01.STR.0000057579.25430.16 12624284

[B11] Yarlagadda S Rajendran P Miss JC Banki NM Kopelnik A Wu AH Ko N Gelb AW Lawton MT Smith WS Young WL Zaroff JG Cardiovascular predictors of in-patient mortality after subarachnoid hemorrhage Neurocrit Care 2006 5 102 107 10.1385/NCC:5:2:102 17099255

[B12] Nakagawa I Kurokawa S Nakase H Hyponatremia is predictable in patients with aneurysmal subarachnoid hemorrhage – clinical significance of serum atrial natriuretic peptide Acta Neurochir (Wien) 2010 152 2147 2152 10.1007/s00701-010-0735-1 20680650

[B13] McGirt MJ Lynch JR Blessing R Warner DS Friedman AH Laskowitz DT Serum von Willebrand factor, matrix metalloproteinase-9, and vascular endothelial growth factor levels predict the onset of cerebral vasospasm after aneurysmal subarachnoid hemorrhage Neurosurgery 2002 51 1128 1134 discussion 1134–1135 10.1097/00006123-200211000-00005 12383357

[B14] Fergusen S Macdonald RL Predictors of cerebral infarction in patients with aneurysmal subarachnoid hemorrhage Neurosurgery 2007 60 658 667 discussion 667 17415202 10.1227/01.NEU.0000255396.23280.31

[B15] Rosengart AJ Schultheiss KE Tolentino J Macdonald RL Prognostic factors for outcome in patients with aneurysmal subarachnoid hemorrhage Stroke 2007 38 2315 2321 10.1161/STROKEAHA.107.484360 17569871

[B16] Liszczak TM Varsos VG Black PM Kistler JP Zervas NT Cerebral arterial constriction after experimental subarachnoid hemorrhage is associated with blood components within the arterial wall J Neurosurg 1983 58 18 26 10.3171/jns.1983.58.1.0018 6847905

[B17] Takemae T Branson PJ Alksne JF Intimal proliferation of cerebral arteries after subarachnoid blood injection in pigs J Neurosurg 1984 61 494 500 10.3171/jns.1984.61.3.0494 6747685

[B18] Macdonald RL Pluta RM Zhang JH Cerebral vasospasm after subarachnoid hemorrhage: the emerging revolution Nat Clin Pract Neurol 2007 3 256 263 17479073 10.1038/ncpneuro0490

[B19] Dhar R Diringer MN The burden of the systemic inflammatory response predicts vasospasm and outcome after subarachnoid hemorrhage Neurocrit Care 2008 8 404 412 10.1007/s12028-008-9054-2 18196475 PMC2538678

[B20] Lad SP Hegen H Gupta G Deisenhammer F Steinberg GK Proteomic biomarker discovery in cerebrospinal fluid for cerebral vasospasm following subarachnoid hemorrhage J Stroke Cerebrovasc Dis 2012 21 30 41 10.1016/j.jstrokecerebrovasdis.2010.04.004 20851633

[B21] Fassbender K Ries S Schminke U Schneider S Hennerici M Inflammatory cytokines in CSF in bacterial meningitis: association with altered blood flow velocities in basal cerebral arteries J Neurol Neurosurg Psychiatry 1996 61 57 61 10.1136/jnnp.61.1.57 8676162 PMC486459

[B22] Peterson JW Kwun BD Teramura A Hackett JD Morgan JA Nishizawa S Bun T Zervas NT Immunological reaction against the aging human subarachnoid erythrocyte, A model for the onset of cerebral vasospasm after subarachnoid hemorrhage J Neurosurg 1989 71 5 Pt 1 718 726 2809726 10.3171/jns.1989.71.5.0718

[B23] Kasuya H Shimizu T Activated complement components C3a and C4a in cerebrospinal fluid and plasma following subarachnoid hemorrhage J Neurosurg 1989 71 5 Pt 1 741 746 2809729 10.3171/jns.1989.71.5.0741

[B24] Schoch B Regel JP Wichert M Gasser T Volbracht L Stolke D Analysis of intrathecal interleukin-6 as a potential predictive factor for vasospasm in subarachnoid hemorrhage Neurosurgery 2007 60 828 836 discussion 828–836 17460517 10.1227/01.NEU.0000255440.21495.80

[B25] Sarrafzadeh A Schlenk F Gericke C Vajkoczy P Relevance of cerebral interleukin-6 after aneurysmal subarachnoid hemorrhage Neurocrit Care 2010 13 339 346 10.1007/s12028-010-9432-4 20725805

[B26] Levin ER Gardner DG Samson WK Natriuretic peptides N Engl J Med 1998 339 321 328 10.1056/NEJM199807303390507 9682046

[B27] Taub PR Fields JD Wu AH Miss JC Lawton MT Smith WS Young WL Zaroff JG Ko NU Elevated BNP is associated with vasospasm-independent cerebral infarction following aneurysmal subarachnoid hemorrhage Neurocrit Care 2011 15 13 18 10.1007/s12028-011-9535-6 21479679 PMC3133817

[B28] Zakynthinos E Kiropoulos T Gourgoulianis K Filippatos G Diagnostic and prognostic impact of brain natriuretic peptide in cardiac and noncardiac diseases Heart Lung 2008 37 275 285 10.1016/j.hrtlng.2007.05.010 18620103

[B29] de Bold AJ Cardiac natriuretic peptides gene expression and secretion in inflammation J Investig Med 2009 57 29 32 19158604 10.2310/JIM.0b013e3181948b37

[B30] Berendes E Walter M Cullen P Prien T Van Aken H Horsthemke J Schulte M von Wild K Scherer R Secretion of brain natriuretic peptide in patients with aneurysmal subarachnoid haemorrhage Lancet 1997 349 245 249 10.1016/S0140-6736(96)08093-2 9014912

[B31] Tomida M Muraki M Uemura K Yamasaki K Plasma concentrations of brain natriuretic peptide in patients with subarachnoid hemorrhage Stroke 1998 29 1584 1587 10.1161/01.STR.29.8.1584 9707197

[B32] Sviri GE Shik V Raz B Soustiel JF Role of brain natriuretic peptide in cerebral vasospasm Acta Neurochir (Wien) 2003 145 851 860 discussion 860 10.1007/s00701-003-0101-7 14577006

[B33] Wysocki SJ Zheng MH Smith A Norman PE Vascular endothelial growth factor (VEGF) expression during arterial repair in the pig Eur J Vasc Endovasc Surg 1998 15 225 230 10.1016/S1078-5884(98)80180-9 9587335

[B34] Martin J Learning from vascular remodelling Clin Exp Allergy 2000 30 Suppl 1 33 36 10849472 10.1046/j.1365-2222.2000.00094.x

[B35] Bergers G Brekken R McMahon G Vu TH Itoh T Tamaki K Tanzawa K Thorpe P Itohara S Werb Z Hanahan D Matrix metalloproteinase-9 triggers the angiogenic switch during carcinogenesis Nat Cell Biol 2000 2 737 744 10.1038/35036374 11025665 PMC2852586

[B36] Zempo N Koyama N Kenagy RD Lea HJ Clowes AW Regulation of vascular smooth muscle cell migration and proliferation in vitro and in injured rat arteries by a synthetic matrix metalloproteinase inhibitor Arterioscler Thromb Vasc Biol 1996 16 28 33 8548422 10.1161/01.atv.16.1.28

[B37] Adner M Jansen I Edvinsson L Endothelin-A receptors mediate contraction in human cerebral, meningeal and temporal arteries J Auton Nerv Syst 1994 49 Suppl S117 S121 7836667 10.1016/0165-1838(94)90098-1

[B38] Suzuki K Meguro K Sakurai T Saitoh Y Takeuchi S Nose T Endothelin-1 concentration increases in the cerebrospinal fluid in cerebral vasospasm caused by subarachnoid hemorrhage Surg Neurol 2000 53 131 135 10.1016/S0090-3019(99)00179-2 10713190

[B39] Kastner S Oertel MF Scharbrodt W Krause M Boker DK Deinsberger W Endothelin-1 in plasma, cisternal CSF and microdialysate following aneurysmal SAH Acta Neurochir (Wien) 2005 147 1271 1279 discussion 1279 10.1007/s00701-005-0633-0 16193351

[B40] Mascia L Fedorko L Stewart DJ Mohamed F TerBrugge K Ranieri VM Wallace MC Temporal relationship between endothelin-1 concentrations and cerebral vasospasm in patients with aneurysmal subarachnoid hemorrhage Stroke 2001 32 1185 1190 10.1161/01.STR.32.5.1185 11340231

[B41] Zanier ER Refai D Zipfel GJ Zoerle T Longhi L Esparza TJ Spinner ML Bateman RJ Brody DL Stocchetti N Neurofilament light chain levels in ventricular cerebrospinal fluid after acute aneurysmal subarachnoid haemorrhage J Neurol Neurosurg Psychiatry 2011 82 157 159 10.1136/jnnp.2009.177667 20571038 PMC3716281

[B42] Guo J Shi Z Yang K Tian JH Jiang L Endothelin receptor antagonists for subarachnoid hemorrhage Cochrane Database Syst Rev 2012 9 CD008354 22972119 10.1002/14651858.CD008354.pub2 PMC11513183

[B43] Sills AK Jr Clatterbuck RE Thompson RC Cohen PL Tamargo RJ Endothelial cell expression of intercellular adhesion molecule 1 in experimental posthemorrhagic vasospasm Neurosurgery 1997 41 453 460 discussion 460–461 10.1097/00006123-199708000-00025 9257314

[B44] Nissen JJ Mantle D Gregson B Mendelow AD Serum concentration of adhesion molecules in patients with delayed ischaemic neurological deficit after aneurysmal subarachnoid haemorrhage: the immunoglobulin and selectin superfamilies J Neurol Neurosurg Psychiatry 2001 71 329 333 10.1136/jnnp.71.3.329 11511705 PMC1737572

[B45] Oshiro EM Hoffman PA Dietsch GN Watts MC Pardoll DM Tamargo RJ Inhibition of experimental vasospasm with anti-intercellular adhesion molecule-1 monoclonal antibody in rats Stroke 1997 28 2031 2037 discussion 2037–2038 10.1161/01.STR.28.10.2031 9341715

[B46] Rothoerl RD Schebesch KM Kubitza M Woertgen C Brawanski A Pina AL ICAM-1 and VCAM-1 expression following aneurysmal subarachnoid hemorrhage and their possible role in the pathophysiology of subsequent ischemic deficits Cerebrovasc Dis 2006 22 143 149 10.1159/000093243 16691023

[B47] Van Geel WJ Rosengren LE Verbeek MM An enzyme immunoassay to quantify neurofilament light chain in cerebrospinal fluid J Immunol Methods 2005 296 179 185 10.1016/j.jim.2004.11.015 15680162

[B48] Petzold A Shaw G Comparison of two ELISA methods for measuring levels of the phosphorylated neurofilament heavy chain J Immunol Methods 2007 319 34 40 10.1016/j.jim.2006.09.021 17140597

[B49] Petzold A Keir G Kay A Kerr M Thompson EJ Axonal damage and outcome in subarachnoid haemorrhage J Neurol Neurosurg Psychiatry 2006 77 753 759 10.1136/jnnp.2005.085175 16705199 PMC2077447

[B50] Pike BR Flint J Dave JR Lu XC Wang KK Tortella FC Hayes RL Accumulation of calpain and caspase-3 proteolytic fragments of brain-derived alphaII-spectrin in cerebral spinal fluid after middle cerebral artery occlusion in rats J Cereb Blood Flow Metab 2004 24 98 106 14688621 10.1097/01.WCB.0000098520.11962.37

[B51] Lewis SB Velat GJ Miralia L Papa L Aikman JM Wolper RA Firment CS Liu MC Pineda JA Wang KK Hayes RL Alpha-II spectrin breakdown products in aneurysmal subarachnoid hemorrhage: a novel biomarker of proteolytic injury J Neurosurg 2007 107 792 796 10.3171/JNS-07/10/0792 17937225

[B52] Donato R S-100 proteins Cell Calcium 1986 7 123 145 10.1016/0143-4160(86)90017-5 3521884

[B53] Rustandi RR Drohat AC Baldisseri DM Wilder PT Weber DJ The Ca(2+)-dependent interaction of S100B(beta beta) with a peptide derived from p53 Biochemistry 1998 37 1951 1960 10.1021/bi972701n 9485322

[B54] Moritz S Warnat J Bele S Graf BM Woertgen C The prognostic value of NSE and S100B from serum and cerebrospinal fluid in patients with spontaneous subarachnoid hemorrhage J Neurosurg Anesthesiol 2010 22 21 31 10.1097/ANA.0b013e3181bdf50d 20027011

[B55] Siman R Giovannone N Toraskar N Frangos S Stein SC Levine JM Kumar MA Evidence that a panel of neurodegeneration biomarkers predicts vasospasm, infarction, and outcome in aneurysmal subarachnoid hemorrhage PLoS One 2011 6 e28938 10.1371/journal.pone.0028938 22174930 PMC3235169

[B56] Goyal A Failla MD Niyonkuru C Amin K Fabio A Berger RP Wagner AK S100b as a prognostic biomarker in outcome prediction for patients with severe traumatic brain injury J Neurotrauma 2013 30 946 957 10.1089/neu.2012.2579 23190274 PMC3684103

[B57] Pelinka LE Kroepfl A Leixnering M Buchinger W Raabe A Redl H GFAP versus S100B in serum after traumatic brain injury: relationship to brain damage and outcome J Neurotrauma 2004 21 1553 1561 10.1089/neu.2004.21.1553 15684648

[B58] Vos PE Lamers KJ Hendriks JC van Haaren M Beems T Zimmerman C van Geel W de Reus H Biert J Verbeek MM Glial and neuronal proteins in serum predict outcome after severe traumatic brain injury Neurology 2004 62 1303 1310 10.1212/01.WNL.0000120550.00643.DC 15111666

[B59] Guzel A Er U Tatli M Aluclu U Ozkan U Duzenli Y Satici O Guzel E Kemaloglu S Ceviz A Kaplan A Serum neuron-specific enolase as a predictor of short-term outcome and its correlation with Glasgow Coma Scale in traumatic brain injury Neurosurg Rev 2008 31 439 444 discussion 444–445 10.1007/s10143-008-0148-2 18560914

[B60] Papa L Akinyi L Liu MC Pineda JA Tepas JJ 3rd Oli MW Zheng W Robinson G Robicsek SA Gabrielli A Heaton SC Hannay HJ Demery JA Brophy GM Layon J Robertson CS Hayes RL Wang KK Ubiquitin C-terminal hydrolase is a novel biomarker in humans for severe traumatic brain injury Crit Care Med 2010 38 138 144 10.1097/CCM.0b013e3181b788ab 19726976 PMC3445330

[B61] Brophy GM Mondello S Papa L Robicsek SA Gabrielli A Tepas J 3rd Buki A Robertson C Tortella FC Hayes RL Wang KK Biokinetic analysis of ubiquitin C-terminal hydrolase-L1 (UCH-L1) in severe traumatic brain injury patient biofluids J Neurotrauma 2011 28 861 870 10.1089/neu.2010.1564 21309726 PMC3113451

[B62] Mondello S Akinyi L Buki A Robicsek S Gabrielli A Tepas J Papa L Brophy GM Tortella F Hayes RL Wang KK Clinical utility of serum levels of ubiquitin C-terminal hydrolase as a biomarker for severe traumatic brain injury Neurosurgery 2012 70 666 675 21937927 10.1227/NEU.0b013e318236a809 PMC3288385

[B63] Thomas DG Palfreyman JW Ratcliffe JG Serum-myelin-basic-protein assay in diagnosis and prognosis of patients with head injury Lancet 1978 1 113 115 87549 10.1016/s0140-6736(78)90415-4

[B64] Yamazaki Y Yada K Morii S Kitahara T Ohwada T Diagnostic significance of serum neuron-specific enolase and myelin basic protein assay in patients with acute head injury Surg Neurol 1995 43 267 270 discussion 270–271 10.1016/0090-3019(95)80012-6 7540773

[B65] Liliang PC Liang CL Weng HC Lu K Wang KW Chen HJ Chuang JH Tau proteins in serum predict outcome after severe traumatic brain injury J Surg Res 2010 160 302 307 10.1016/j.jss.2008.12.022 19345376

[B66] Pineda JA Lewis SB Valadka AB Papa L Hannay HJ Heaton SC Demery JA Liu MC Aikman JM Akle V Brophy GM Tepas JJ Wang KK Robertson CS Hayes RL Clinical significance of alphaII-spectrin breakdown products in cerebrospinal fluid after severe traumatic brain injury J Neurotrauma 2007 24 354 366 10.1089/neu.2006.003789 17375999

[B67] Mondello S Robicsek SA Gabrielli A Brophy GM Papa L Tepas J Robertson C Buki A Scharf D Jixiang M Akinyi L Muller U Wang KK Hayes RL alphaII-spectrin breakdown products (SBDPs): diagnosis and outcome in severe traumatic brain injury patients J Neurotrauma 2010 27 1203 1213 10.1089/neu.2010.1278 20408766 PMC2942904

[B68] Ost M Nylen K Csajbok L Ohrfelt AO Tullberg M Wikkelso C Nellgard P Rosengren L Blennow K Nellgard B Initial CSF total tau correlates with 1-year outcome in patients with traumatic brain injury Neurology 2006 67 1600 1604 10.1212/01.wnl.0000242732.06714.0f 17101890

[B69] Zetterberg H Smith DH Blennow K Biomarkers of mild traumatic brain injury in cerebrospinal fluid and blood Nat Rev Neurol 2013 9 201 210 10.1038/nrneurol.2013.9 23399646 PMC4513656

[B70] Ciccarelli R Di Iorio P Bruno V Battaglia G D'Alimonte I D'Onofrio M Nicoletti F Caciagli F Activation of A(1) adenosine or mGlu3 metabotropic glutamate receptors enhances the release of nerve growth factor and S-100beta protein from cultured astrocytes Glia 1999 27 275 281 10.1002/(SICI)1098-1136(199909)27:3<275::AID-GLIA9>3.0.CO;2-0 10457374

[B71] Whitaker-Azmitia PM Murphy R Azmitia EC Stimulation of astroglial 5-HT1A receptors releases the serotonergic growth factor, protein S-100, and alters astroglial morphology Brain Res 1990 528 155 158 10.1016/0006-8993(90)90210-3 2245332

[B72] Suzuki F Kato K Kato T Ogasawara N S-100 protein in clonal astroglioma cells is released by adrenocorticotropic hormone and corticotropin-like intermediate-lobe peptide J Neurochem 1987 49 1557 1563 10.1111/j.1471-4159.1987.tb01027.x 2822856

[B73] Elting JW de Jager AE Teelken AW Schaaf MJ Maurits NM van der Naalt J Sibinga CT Sulter GA De Keyser J Comparison of serum S-100 protein levels following stroke and traumatic brain injury J Neurol Sci 2000 181 104 110 10.1016/S0022-510X(00)00442-1 11099719

[B74] Muller K Townend W Biasca N Unden J Waterloo K Romner B Ingebrigtsen T S100B serum level predicts computed tomography findings after minor head injury J Trauma 2007 62 1452 1456 10.1097/TA.0b013e318047bfaa 17563665

[B75] Zongo D Ribereau-Gayon R Masson F Laborey M Contrand B Salmi LR Montaudon D Beaudeux JL Meurin A Dousset V Loiseau H Lagarde E S100-B protein as a screening tool for the early assessment of minor head injury Ann Emerg Med 2012 59 209 218 10.1016/j.annemergmed.2011.07.027 21944878

[B76] Rothoerl RD Woertgen C High serum S100B levels for trauma patients without head injuries Neurosurgery 2001 49 1490 1491 author reply 1492–1493 10.1097/00006123-200112000-00054 11859837

[B77] Marangos PJ Schmechel DE Neuron specific enolase, a clinically useful marker for neurons and neuroendocrine cells Annu Rev Neurosci 1987 10 269 295 10.1146/annurev.ne.10.030187.001413 3551759

[B78] Ross SA Cunningham RT Johnston CF Rowlands BJ Neuron-specific enolase as an aid to outcome prediction in head injury Br J Neurosurg 1996 10 471 476 10.1080/02688699647104 8922706

[B79] Pelinka LE Hertz H Mauritz W Harada N Jafarmadar M Albrecht M Redl H Bahrami S Nonspecific increase of systemic neuron-specific enolase after trauma: clinical and experimental findings Shock 2005 24 119 123 10.1097/01.shk.0000168876.68154.43 16044081

[B80] Jackson P Thompson RJ The demonstration of new human brain-specific proteins by high-resolution two-dimensional polyacrylamide gel electrophoresis J Neurol Sci 1981 49 429 438 10.1016/0022-510X(81)90032-0 7217993

[B81] Tongaonkar P Chen L Lambertson D Ko B Madura K Evidence for an interaction between ubiquitin-conjugating enzymes and the 26S proteasome Mol Cell Biol 2000 20 4691 4698 10.1128/MCB.20.13.4691-4698.2000 10848595 PMC85887

[B82] Papa L Lewis LM Silvestri S Falk JL Giordano P Brophy GM Demery JA Liu MC Mo J Akinyi L Mondello S Schmid K Robertson CS Tortella FC Hayes RL Wang KK Serum levels of ubiquitin C-terminal hydrolase distinguish mild traumatic brain injury from trauma controls and are elevated in mild and moderate traumatic brain injury patients with intracranial lesions and neurosurgical intervention J Trauma Acute Care Surg 2012 72 335 1344 10.1097/TA.0b013e3182491e3d PMC5516044 22673263

[B83] Missler U Wiesmann M Wittmann G Magerkurth O Hagenstrom H Measurement of glial fibrillary acidic protein in human blood: analytical method and preliminary clinical results Clin Chem 1999 45 138 141 9895354

[B84] Lumpkins KM Bochicchio GV Keledjian K Simard JM McCunn M Scalea T Glial fibrillary acidic protein is highly correlated with brain injury J Trauma 2008 65 778 782 discussion 782–784 10.1097/TA.0b013e318185db2d 18849790

[B85] Pelinka LE Kroepfl A Schmidhammer R Krenn M Buchinger W Redl H Raabe A Glial fibrillary acidic protein in serum after traumatic brain injury and multiple trauma J Trauma 2004 57 1006 1012 10.1097/01.TA.0000108998.48026.C3 15580024

[B86] Papa L Lewis LM Falk JL Zhang Z Silvestri S Giordano P Brophy GM Demery JA Dixit NK Ferguson I Liu MC Mo J Akinyi L Schmid K Mondello S Robertson CS Tortella FC Hayes RL Wang KK Elevated levels of serum glial fibrillary acidic protein breakdown products in mild and moderate traumatic brain injury are associated with intracranial lesions and neurosurgical intervention Ann Emerg Med 2012 59 471 483 10.1016/j.annemergmed.2011.08.021 22071014 PMC3830977

[B87] Okonkwo DO Yue JK Puccio AM Panczykowski DM Inoue T McMahon PJ Sorani MD Yuh EL Lingsma HF Maas AI Valadka AB Manley GT Casey SS Cheong M Cooper SR Dams-O'Connor K Gordon WA Hricik AJ Hochberger K Menon DK Mukherjee P Sinha TK Schnyer DM Vassar MJ Transforming, Research Clinical Knowledge In Traumatic Brain Injury Investigators GFAP-BDP as an acute diagnostic marker in traumatic brain injury: results from the prospective transforming research and clinical knowledge in traumatic brain injury study J Neurotrauma 2013 30 1490 1497 10.1089/neu.2013.2883 23489259 PMC3751263

[B88] Honda M Tsuruta R Kaneko T Kasaoka S Yagi T Todani M Fujita M Izumi T Maekawa T Serum glial fibrillary acidic protein is a highly specific biomarker for traumatic brain injury in humans compared with S-100B and neuron-specific enolase J Trauma 2010 69 104 109 10.1097/TA.0b013e3181bbd485 20093985

[B89] Jauch EC Lindsell C Broderick J Fagan SC Tilley BC Levine SR Association of serial biochemical markers with acute ischemic stroke: the National Institute of Neurological Disorders and Stroke recombinant tissue plasminogen activator Stroke Study Stroke 2006 37 2508 2513 10.1161/01.STR.0000242290.01174.9e 16960091

[B90] Foerch C Singer OC Neumann-Haefelin T Du Mesnil De Rochemont R Steinmetz H Sitzer M Evaluation of serum S100B as a surrogate marker for long-term outcome and infarct volume in acute middle cerebral artery infarction Arch Neurol 2005 62 1130 1134 10.1001/archneur.62.7.1130 16009772

[B91] Dassan P Keir G Brown MM Criteria for a clinically informative serum biomarker in acute ischaemic stroke: a review of S100B Cerebrovasc Dis 2009 27 295 302 10.1159/000199468 19202335

[B92] Jonsson H Johnsson P Birch-Iensen M Alling C Westaby S Blomquist S S100B as a predictor of size and outcome of stroke after cardiac surgery Ann Thorac Surg 2001 71 1433 1437 10.1016/S0003-4975(00)02612-6 11383778

[B93] Ahmad O Wardlaw J Whiteley WN Correlation of levels of neuronal and glial markers with radiological measures of infarct volume in ischaemic stroke: a systematic review Cerebrovasc Dis 2012 33 47 54 10.1159/000332810 22133844

[B94] Hill MD Jackowski G Bayer N Lawrence M Jaeschke R Biochemical markers in acute ischemic stroke CMAJ 2000 162 1139 1140 10789628 PMC1232364

[B95] Foerch C Otto B Singer OC Neumann-Haefelin T Yan B Berkefeld J Steinmetz H Sitzer M Serum S100B predicts a malignant course of infarction in patients with acute middle cerebral artery occlusion Stroke 2004 35 2160 2164 10.1161/01.STR.0000138730.03264.ac 15297628

[B96] Foerch C Wunderlich MT Dvorak F Humpich M Kahles T Goertler M Alvarez-Sabin J Wallesch CW Molina CA Steinmetz H Sitzer M Montaner J Elevated serum S100B levels indicate a higher risk of hemorrhagic transformation after thrombolytic therapy in acute stroke Stroke 2007 38 2491 2495 10.1161/STROKEAHA.106.480111 17673718

[B97] Kazmierski R Michalak S Wencel-Warot A Nowinski WL Serum tight-junction proteins predict hemorrhagic transformation in ischemic stroke patients Neurology 2012 79 1677 1685 10.1212/WNL.0b013e31826e9a83 22993287

[B98] Saenger AK Christenson RH Stroke biomarkers: progress and challenges for diagnosis, prognosis, differentiation, and treatment Clin Chem 2010 56 21 33 10.1373/clinchem.2009.133801 19926776

[B99] Yoo JH Lee SC Elevated levels of plasma homocyst(e)ine and asymmetric dimethylarginine in elderly patients with stroke Atherosclerosis 2001 158 425 430 10.1016/S0021-9150(01)00444-0 11583722

[B100] Leong T Zylberstein D Graham I Lissner L Ward D Fogarty J Bengtsson C Bjorkelund C Thelle D Asymmetric dimethylarginine independently predicts fatal and nonfatal myocardial infarction and stroke in women: 24-year follow-up of the population study of women in Gothenburg Arterioscler Thromb Vasc Biol 2008 28 961 967 10.1161/ATVBAHA.107.156596 18292394

[B101] Pikula A Boger RH Beiser AS Maas R DeCarli C Schwedhelm E Himali JJ Schulze F Au R Kelly-Hayes M Kase CS Vasan RS Wolf PA Seshadri S Association of plasma ADMA levels with MRI markers of vascular brain injury: Framingham offspring study Stroke 2009 40 2959 2964 10.1161/STROKEAHA.109.557116 19644064 PMC2749945

[B102] Clark AW Krekoski CA Bou SS Chapman KR Edwards DR Increased gelatinase A (MMP-2) and gelatinase B (MMP-9) activities in human brain after focal ischemia Neurosci Lett 1997 238 53 56 10.1016/S0304-3940(97)00859-8 9464653

[B103] Alvarez-Sabin J Delgado P Abilleira S Molina CA Arenillas J Ribo M Santamarina E Quintana M Monasterio J Montaner J Temporal profile of matrix metalloproteinases and their inhibitors after spontaneous intracerebral hemorrhage: relationship to clinical and radiological outcome Stroke 2004 35 1316 1322 10.1161/01.STR.0000126827.69286.90 15087562

[B104] Montaner J Alvarez-Sabin J Molina C Angles A Abilleira S Arenillas J Gonzalez MA Monasterio J Matrix metalloproteinase expression after human cardioembolic stroke: temporal profile and relation to neurological impairment Stroke 2001 32 1759 1766 10.1161/01.STR.32.8.1759 11486102

[B105] Montaner J Alvarez-Sabin J Molina CA Angles A Abilleira S Arenillas J Monasterio J Matrix metalloproteinase expression is related to hemorrhagic transformation after cardioembolic stroke Stroke 2001 32 2762 2767 10.1161/hs1201.99512 11739970

[B106] Montaner J Molina CA Monasterio J Abilleira S Arenillas JF Ribo M Quintana M Alvarez-Sabin J Matrix metalloproteinase-9 pretreatment level predicts intracranial hemorrhagic complications after thrombolysis in human stroke Circulation 2003 107 598 603 10.1161/01.CIR.0000046451.38849.90 12566373

[B107] Rosell A Alvarez-Sabin J Arenillas JF Rovira A Delgado P Fernandez-Cadenas I Penalba A Molina CA Montaner J A matrix metalloproteinase protein array reveals a strong relation between MMP-9 and MMP-13 with diffusion-weighted image lesion increase in human stroke Stroke 2005 36 1415 1420 10.1161/01.STR.0000170641.01047.cc 15947272

[B108] Gappoeva MU Izykenova GA Granstrem OK Dambinova SA Expression of NMDA neuroreceptors in experimental ischemia Biochemistry (Mosc) 2003 68 696 702 10.1023/A:1024678112357 12943515

[B109] Dambinova SA Bettermann K Glynn T Tews M Olson D Weissman JD Sowell RL Diagnostic potential of the NMDA receptor peptide assay for acute ischemic stroke PLoS One 2012 7 e42362 10.1371/journal.pone.0042362 22848761 PMC3407099

[B110] Dambinova SA Khounteev GA Izykenova GA Zavolokov IG Ilyukhina AY Skoromets AA Blood test detecting autoantibodies to N-methyl-D-aspartate neuroreceptors for evaluation of patients with transient ischemic attack and stroke Clin Chem 2003 49 1752 1762 10.1373/49.10.1752 14500616

[B111] Bokesch PM Izykenova GA Justice JB Easley KA Dambinova SA NMDA receptor antibodies predict adverse neurological outcome after cardiac surgery in high-risk patients Stroke 2006 37 1432 1436 10.1161/01.STR.0000221295.14547.c8 16627793

[B112] Herrmann M Vos P Wunderlich MT de Bruijn CH Lamers KJ Release of glial tissue-specific proteins after acute stroke: a comparative analysis of serum concentrations of protein S-100B and glial fibrillary acidic protein Stroke 2000 31 2670 2677 10.1161/01.STR.31.11.2670 11062293

[B113] Foerch C Curdt I Yan B Dvorak F Hermans M Berkefeld J Raabe A Neumann-Haefelin T Steinmetz H Sitzer M Serum glial fibrillary acidic protein as a biomarker for intracerebral haemorrhage in patients with acute stroke J Neurol Neurosurg Psychiatry 2006 77 181 184 10.1136/jnnp.2005.074823 16174653 PMC2077601

[B114] Dvorak F Haberer I Sitzer M Foerch C Characterisation of the diagnostic window of serum glial fibrillary acidic protein for the differentiation of intracerebral haemorrhage and ischaemic stroke Cerebrovasc Dis 2009 27 37 41 10.1159/000172632 19018136

[B115] Unden J Strandberg K Malm J Campbell E Rosengren L Stenflo J Norrving B Romner B Lindgren A Andsberg G Explorative investigation of biomarkers of brain damage and coagulation system activation in clinical stroke differentiation J Neurol 2009 256 72 77 10.1007/s00415-009-0054-8 19221847

[B116] Ernst A Suhr J Kohrle J Bergmann A Detection of stable N-terminal protachykinin A immunoreactivity in human plasma and cerebrospinal fluid Peptides 2008 29 1201 1206 10.1016/j.peptides.2008.02.006 18374454

[B117] Doehner W von Haehling S Suhr J Ebner N Schuster A Nagel E Melms A Wurster T Stellos K Gawaz M Bigalke B Elevated plasma levels of neuropeptide proenkephalin a predict mortality and functional outcome in ischemic stroke J Am Coll Cardiol 2012 60 346 354 10.1016/j.jacc.2012.04.024 22813614

[B118] Reynolds MA Kirchick HJ Dahlen JR Anderberg JM McPherson PH Nakamura KK Laskowitz DT Valkirs GE Buechler KF Early biomarkers of stroke Clin Chem 2003 49 1733 1739 10.1373/49.10.1733 14500614

[B119] Laskowitz DT Blessing R Floyd J White WD Lynch JR Panel of biomarkers predicts stroke Ann N Y Acad Sci 2005 1053 30 10.1196/annals.1344.051 16179504

[B120] Laskowitz DT Kasner SE Saver J Remmel KS Jauch EC Clinical usefulness of a biomarker-based diagnostic test for acute stroke: the Biomarker Rapid Assessment in Ischemic Injury (BRAIN) study Stroke 2009 40 77 85 10.1161/STROKEAHA.108.516377 18948614

[B121] Montaner J Mendioroz M Ribo M Delgado P Quintana M Penalba A Chacon P Molina C Fernandez-Cadenas I Rosell A Alvarez-Sabin J A panel of biomarkers including caspase-3 and D-dimer may differentiate acute stroke from stroke-mimicking conditions in the emergency department J Intern Med 2011 270 166 174 10.1111/j.1365-2796.2010.02329.x 21198992

[B122] Doehner W Diagnostic biomarkers in cardiovascular disease: the proteomics approach Eur Heart J 2012 33 2249 2251 10.1093/eurheartj/ehs187 22766582

